# Remarks upon the Different Appearances of the Back, Breast, and Ribs, in Persons Affected with Spinal Diseases; and on the Effects of Spinal Distortion on the Sanguineous Circulation

**Published:** 1820-11

**Authors:** Edward Harrison


FOR THE LONDON MEDICAL AND PHYSICAL JOURNAL.

### *Remarks upon the different Appearances of the Back, Breast, and Ribs, in Persons affected with Spinal Diseases; and on the Effects of Spinal Distortion on the Sanguineous Circulation.* By Edward Harrison, M.D. F.R.A.S. Ed. &c.

THE human system consists of different structures and substances, to fit it for the various occasions of life. Of these, some are for the purpose of locomotion; others, for supplying us with needful food, or removing what is noxious; others again are intended to sustain the weight and preserve the form of the body. The scull-cap is made strong and round, to defend the tender brain. The spinal marrow is guarded and secured by curious mechanism of great thickness, and by strong ligaments, to prevent injury to its delicate texture. For the same reason, the heart, lungs, and contiguous vessels, are placed within a bony case, to protect them from injurious compression in the performance of their complicated operations. The enclosed lungs require a capacious chest for their full expansion, and to permit the blood in them to undergo its proper changes in the pulmonary cells. When they are compressed and impeded in their motions, by the bony frame being squeezed and thrust out of shape, cough, dyspnœa, and dangerous congestions, ensue from slight causes. These lead to the formation of tubercles, to inflammation, suppuration, and fatal consumption.

Unless room be allowed in the chest for the heart freely to admit and expel the blood sent to it, accumulations form in the several cavities. The heart, in struggling to free itself from the oppressive load, is thrown into violent agitations, and thus is laid the foundation of organic derangements in its several cavities, in the valves, and neighbouring blood-vessels.

Crookedness in the dorsal spine not only affects the back, but leads to a derangement of the sternum and ribs, highly prejudicial, in many ways, to the subject of it.

The ribs are firmly joined to the vertebræ, by a double articulation, at one end, and to the sternum at the other. When we examine this curious mechanism, it is discovered immediately that the ribs, fixed and bound by ligamentous attachments, can only be moved upwards and downwards. In this direction they slide gently at every respiration over the thoracic viscera, neither inducing pressure, irritation, nor squeezing. The ribs become very differently affected in respect to these organs, when the vertebræ, forced from their natural places, drag them into new situations. The direction of the ribs being thus changed, the sides of the chest become flatter, or their edges are turned towards the costal pleuræ. The sternum is likewise driven from its original station, in consequence of its connection with the ribs. In this manner the thorax often gets disgustingly mis-shapen.* The fore-part, instead of preserving its beautiful rotundity, becomes peaked, or what is called chicken-breasted. The tops of the shoulders are of unequal height, and project irregularly forward. The right scapula, standing outwards, forms a disagreeable swelling, or tuberosity; while the left, sunk down, is nearly invisible. A posterior curve of the cervical or superior dorsal vertebræ shortens the back, and constitutes that unsightly gibbosity usually denominated the hump-back. Persons so unhappily shaped move about objects of aversion to the timid, and of terror to young wives. Nor is this all: these unfortunate fellow-creatures, feeling themselves exposed to the shafts of ridicule, tormented too with their numerous infirmities, and, disqualified from occupying any useful station, become irritable, peevish, and malevolent. Thus a deformed body is made the habitation of a distempered mind.

"It is not always necessary that, to the disorders which are found in the situation of the bones of the sternum, and of the ribs that are connected therewith, a depraved situation of the vertebræ of the back should also correspond; which is confirmed by my observations, and particularly in a certain woman, and partly in an old man: to which you may add what was observed in a ricketty child, of which the *Act. Nat. Cur.* just now quoted, give the history; and what the celebrated Haller has described from another little child very accurately.

"However, we must confess, with Severinus, that Cardanus has, as in most other things, taught what is true; and even that the vitiated posture of the vertebræ is the much more frequent cause that a perverted situation of the ribs and of the sternum follow, is demonstrated by other of my observations, and by those of others: among which, if you will read one in particular that I have given in the fourth letter, you will at once understand that, not a perverted and incongruous situation of the ribs and sternum was the consequence of a distortion in the spine, but *a perverted situation of the viscera and vessels of the belly* also; and will at the same time conjecture how much, of course, not only the smaller vessels, among which is, in particular, the thoracic duct, but also the greater part of the nerves and other parts of the like kind, must have been disturbed from their natural situations; which considerations, neither time nor place, nor the principal design we had then in view, suffered us to prosecute.

"Moreover, even the celebrated Helwich will teach us how much all the *thoracic viscera were forced into a very narrow*

* See Case I.

*compass* and confined, by the spine being distorted anteriorly, in a certain matron; and the illustrious Haller will inform you how far the *great artery* was removed from its proper seat in another woman, whose spine had been forced into serpentine inflexions, different from what we see in a natural state, by carrying heavy burthens on her back, which was her method of earning her livelihood. And that this kind of life is, at other times, among the external causes of gibbosity, especially in young bodies, is not only demonstrated by reason, but by the observation of the celebrated Nebellius."*

Many functions are occasionally suspended without injury or inconvenience.

Those of the heart and lungs always go on, whether we are awake or asleep. Stop either of them only for a few minutes, and death, which no art can prevent, certainly follows.

Every deviation of the human thorax from its natural form and dimensions occasions, as we have already observed, some impediment to breathing and the circulation of the blood. The vital fluid, in consequence, does not fully undergo its accustomed changes in the lungs; nor is it properly carried through the vessels, to recruit and repair the losses which are constantly taking place in the body.

Of all the maladies which affect the constitution, such as arise from malformation in the bones of the chest and spine are the most distressingly stubborn, because they are occasioned by distortions hitherto deemed incurable, and impede some of the most important functions of living beings. Of course, no two individuals are similarly affected; the curves upon which they depend vary so much in situation, extent, and direction. The neck, back, and loins, are all subject to them. The cervical bones, admitting of great flexion, are most liable to luxations from violent means.† They are also particularly subject to dislocations slowly and gradually introduced, because, for the several purposes of easy motion, their articulating surfaces are smaller, and their connecting ligaments more pliable, than in the back and loins.

We are told by experienced practitioners, that luxations of the dorsal and lumbar vertebræ are impossible under any circumstances. They found their opinion upon the large surfaces by which these bones correspond, the number and thickness of the connecting ligaments, the strength of the muscles employed, the small motion of each vertebra, and the vertical direction of the articulating apophyses. I am ready to admit, that, owing to the protection given, vertebral dislocations seldom appear in these parts; but, that they sometimes occur in the loins from

* Epist. 27, art. 32. Morgagni.

† Boyer, vol. ii. ch. 6.

external violence, I am enabled to assert from my own experience.* I am unacquainted with any case of luxation in the dorsal vertebræ from injuries received, but I have met with many examples from a gradual enlargement of the articulating fibres.† Nature, which cannot bear sudden alterations, is habituated to them gradually and insensibly. An inconsiderable disturbance of the spinal marrow, suddenly produced, totally deranges its substance, though it is not sensibly injured when the changes operate by degrees. Sometimes one vertebra only is affected, sometimes more, by which the magnitude of the curvature is determined. The disposition of the spine becomes lateral, posterior, or anterior, according to circumstances.

1st. When the head, shoulders, and arms, become heavier than the spine is capable of sustaining with impunity, it is encouraged to bend towards the right side, because the great use which is made of the right hand determines the spine to assume that direction in preference to every other. In this manner arises the lateral incurvation, which is by far the most common variety of spinal distortions.‡

2d. The spine never protrudes backwards of itself. When it assumes that deviation, it has been forced into it by some corporeal exertion, in pulling, lifting, or carrying.

3d. The anterior or inward curve from constitutional causes, most commonly shows itself either in the lower cervical and upper dorsal, or in the inferior lumbar, vertebræ. The former protrusion is attended with very distressing and injurious effects upon the lungs and heart. In the latter, the rectum, bladder, and uterus, are thrust out of their natural situations, and prevented from freely performing their respective offices. These sufferings, however great, are by no means the most formidable or distressing. The trying and perplexing difficulties present themselves during the progress of labour. At this time, from the brim and back of the pelvis being forced inwards, the entrance is reduced, so as to arrest the progress of the child, and render the best efforts of the mother unavailing. In many cases of lingering parturition, the obstacles are gradually surmounted by the inherent powers of the female. On this interesting occasion her exertions are unavailing, because the impediment depends upon a fixed and immovable resistance. All that art can perform under such unhappy circumstances, is to relieve the parent by reducing the bulk of her child. A labour thus conducted necessarily causes the death of the infant, to afford the chance of saving an unfortunate mother.

When it depends upon a wasting in the bodies of the verte-

---

* See Case 6th.

† See Cases 2d, 3d, 5th; and Boyer's Lectures on Luxations.

‡ Morgag. Epist.

bræ, these have, after death, been found entirely removed, leaving the cartilages sound and entire. The loss of substance arises from absorption occasioned by the action of abscesses,* aneurisms,† and other tumors upon the bones. The spine, deprived in this manner of its natural support, sinks inwards, and the anterior curve is produced. The column, though unsupported where the vertebræ are wanting, is generally preserved nearly erect by the resistance it meets with from the contiguous viscera. In this form of caries, paraplegia is seldom induced, because no pressure‡ falls upon the cord, as in other cases, to intercept the free and unrestrained operations of the medulla spinalis.

Besides the three species of incurvation mentioned above, single bones, or a few contiguous ones, are apt to protrude a little, and rise with roundish heads above their fellows, in the neck and loins. The dorsal vertebræ generally stand forward in a continued line, and make an elevated ridge, which may include the whole or only part of the bones. These several protrusions, or incomplete luxations, are easily understood, by attending to the varying forms of the spinous processes, their connection with the muscles, and the costal articulations.

A small irregularity in the height and disposition of some particular vertebræ is perceptible, on examination, in most delicate females. This disorderly arrangement and disposition of the component parts of the spinal column, though hitherto overlooked and wholly neglected, are, I am persuaded, of great consequence to future health. The effects of this subluxation, not being distinguishable by the symptoms, have never been traced to their origin in the spine. A very slight and partial compression of the cord, or some of its nerves, will disturb the organs to which they run. If we admit the operation of this cause upon all the vertebræ of the neck, back, and loins, in different persons, we shall be at no loss to account for the almost infinite variety and endless complication of nervous symptoms which harass many individuals through life, and baffle the most eminent of the faculty. When we take into account the number, the size, and the distribution of the spinal nerves among the viscera and muscles, we are led to conclude that scarcely a complaint can arise in which they do not participate. Even tetanus, in several instances, has been lately traced to this fertile source of human miseries. But the affections of the nervous system display such a wide field for enquiry and discussion, that we must defer their further consideration to another opportunity, and confine ourselves at present to the

* Morgag. Lieutaud.

† Ibid. Ep. xvii. art. 17; Ep. xxi. art. 17.

‡ Lieut. vol. ii. lib. 4; Lecat, Blanchard, Hildanus.

vascular system. In recent cases these subluxations are easily replaced: parents will therefore best consult the health and comfort of their children by frequently examining the spinal column, and taking the earliest opportunity of counteracting its defects.

I am aware that the foregoing explanation of the origin of spinal incurvations, is at variance with former opinions. Glisson and others contend, that the bones being unequally nourished, and one side growing faster than the other, a curvature is produced by the different thickness of the two sides. In this manner, according to them, arises every deviation of the spine from its natural figure. The extent and magnitude of the projection depends, of course, upon the number of the affected bones. Thus, the back becomes bent because the vertebræ are unequally nourished, and their sides of different degrees of thickness. If we admit that a single vertebra increases in this manner, it is difficult to believe that many of them could be brought to participate in this same irregularity; yet, without admitting a common process to be going on among the bones, I cannòt understand how the extensive curvatures which we daily witness could have been produced. Still, with these admissions, the difficulties are not by any means removed, because a counter-curvature is always formed in another part of the column, to preserve the centre of gravity. In such cases, the exuberant growth must necessarily take place on opposite sides of the spine: otherwise, according to this doctrine, the two curves could not be growing together; and it surely requires more than an ordinary share of credulity to suppose that two such extraordinary processes should be acting in opposition to each other, at the same time, in different parts of the spine. The second opinion, originally proposed by Mayow, leads to a belief that one set of muscles, acting more vigorously than another, bend or draw the bones out of their proper stations, and induce the curves which take place. We have, in the extremities, flexors and extensors to move the limbs in different ways. These, from their employment, have been denominated antagonist muscles; but, to suppose that a struggle takes place between them, is, in my judgment, to entertain a very erroneous idea of what really does occur in the animal economy. When not called into action by the will, they remain quiescent; and, when movements require to be performed, certain muscles are brought into play to effect the intended purpose, but no more are actively employed than the occasion demands; the rest are wholly inactive and unoccupied: no conflicting struggles or counterpoising exertions ever take place among them.

Were it to be proved that in the extremities two sets of muscles are acting in constant opposition to each other, it would not follow in course that those in the back are similarly employed: indeed, there is no such provision, as far as I know, in the structure of the back. The muscles situated there are most of them too large to be concerned in forming the two lateral or sigmoid flexions of the spine, without supposing them to produce a different action at one end from what they do at the other. Such an admission is altogether improbable, and is in direct opposition to every principle of muscular motion in other parts of the human body. We have strong muscles to raise and bend the trunk, but they proceed in unison with one another, and are never placed in opposition. These two motions, the principal ones of which the spine is susceptible, are not synchronous: they interchange with each other, and consequently do not interfere. The muscles destined to move the vertebræ are attached to the articulating fibrous structure. This is stretched by them, and in weakly habits becomes preternaturally elongated, partly by the muscular force pulling it, partly by the bones being pushed against it in the various turns and gesticulations of the body. As these parts gradually give way, the joints slowly enlarge, and admit of greater motion. A slight luxation is first produced in one joint, and afterwards in several. If the further progress of the complaint be arrested in this early stage, the person is said to have got round shoulders, or to have acquired an awkward carriage; but the real state of the spinal column, on which these appearances depend, is not in the least suspected.

Were spinal distortions to originate in the muscles, as some experienced practitioners affirm, we should find them most prevalent among the agricultural youth of both sexes, who are employed to carry heavy burthens and perform the most laborious work; yet it is well known that the disorder seldom appears among them, and, when it does show itself, it can be traced to some obvious cause.

The practice to which this principle leads, is inefficacious and injudicious. The sufferer, placed upon an inclined plane, is confined, and prevented from sliding downwards by a band fixed under the chin. In this situation he is directed to work and twist the muscles of his back, to increase their tone and action. But let us here stop to enquire, whether an individual has been made straight by this operation, protracted as it has been to five, six, and more years?

Has the vertebral column been restored, in a single instance, to its natural shape during this treatment? Has the cure been rendered more perfect, or been finished in less time, with these auxiliaries, than by adopting the horizontal position and resting contrivances of Mr. Baynton? According to my observations, the result has always been unfavourable, both in time and in all other respects. Nor should this excite surprise: the disorder is situated in the articulating structure below the muscles; their motions, at every turn and change, necessarily agitate and disturb the vertebral joints, through their tendinous attachments. Thus the cure is impeded, rendered less certain, and less perfect, than if it had been restricted to rest alone.

Highly as I approve of Mr. Baynton's plan, in taking off the incumbent weight, in giving the muscles and tendinous fibres an opportunity to adapt themselves to their new situations, I think it is extremely defective, because it encourages no trials to replace the luxated vertebræ in their former positions. It therefore leaves the patient in a state of deformity, and, as an unavoidable consequence of it, in bad health. In all other dislocations, whether great or small, practitioners endeavour, in the first instance, to replace the bones, and never think their duty performed until they have fully accomplished their object. When a failure takes place in either extremity, the joint remains weak, deformed, and inadequate to its appointed offices. If, then, such distressing inconveniences spring out of a single dislocation in the limbs, may we not reasonably suppose that disadvantages equally numerous and incapacitating will arise from the luxation of any one of the vertebræ? They certainly are not immediately concerned with locomotion, but they afford a bony tube for the safe custody of the spinal marrow, and canals for the easy passage of its nerves; they give support to the abdominal and thoracic viscera, and room for the performance of their important functions in the living body. Surely offices like these deserve to be guarded and protected with the utmost vigilance. Happily, these vertebral dislocations from internal causes may be easily removed in every recent, and in many old cases, so as to leave no traces in the appearance of the back or in the health of the individual.* I do not mean to include in this statement those extraordinary dislocations in which the vertebræ are flexible and yielding. They have, in some gibbous persons, been found soft enough to be moulded like wax.† This disposition generally takes place at an early age. Between it and the healthy state are several grades, which have not been marked with sufficient care. It is however of importance to observe, that softness is distinct from caries, and must not be confounded with it.

The luxated vertebræ have in some old cases been found united together, and consolidated into an irregular and indistinct mass.‡ Such occurrences have been met with in practice,

---

* See Cases 2d, 3d; also the rest. † Ep. xxvii. art. 33, Hildan. Observat.

‡ Morg. Ep. lxvi. art. 37; Fallop. *de Lux. et Fract. Ossib.*; Ruysch *Observ. Anatom. Chirurg.*; Lieut. vol. ii.

and are upon record; but they take place much less frequently than some of our countrymen have been led to imagine. We must not suffer ourselves to be deceived by them into an hasty belief that the bones are generally diseased, and that therefore nothing effectual can be done for the removal of spinal distortions. Such an erroneous conclusion would paralyze our efforts in the cause of humanity, and lead us to perpetuate the misery of our fellow-creatures, by neglecting the means of relief on every occasion. A disturbance in the circulating system occasioned by deformity, leads to many kinds of indisposition, as the offending cause varies in situation, in extent, and in degree. Luxations in the cervical vertebræ, by impeding the current of blood in the carotids and vertebral arteries, occasion, in many instances, a diminished quantity of it to flow to the head. A scanty supply of arterial blood must necessarily have great influence upon the operations of the mind and body,* because the generation and uniform production of nervous power, the chief regulating principle of animal life, is greatly dependant upon the constant renewal and successive applications of the vital fluid to the brain. From this deficiency we may reasonably conclude, that, as the body will be debilitated, the intellectual faculties will also be weakened in their perceptions and determinations, as well as in the exercise of them, by this state and condition of the blood within the skull. Ignorant as we are of the nature of the human mind, and of its mysterious connection with the corporeal frame, every day's experience shows that a very intimate communication is kept up between them. To this intercourse the brain forms the most immediate and essential link. No wonder then that the dispositions, the affections, and the operations of the mind, should be to a certain degree under its influence. The quantity of blood destined to nourish the lower part of the body or some particular organ, may in like manner be diminished by the displaced vertebræ in the back and loins pressing upon the descending aorta, or its ramifying branches. From the nutritious materials being in this way withdrawn, extenuation and emaciation take place in all the parts from which they are withheld. The scanty supply of blood to the abdominal organs must necessarily interfere with their complicated operations, and lead to the establishment of various diseases. A deficiency of it in the uterus may be reasonably expected to suspend its periodical movements, and induce amenorrhœa in single, and sterility in married, women.

Matters are differently circumstanced above the obstruction. The action of the heart and pulsatory motion of the artery

* Broussais *Journal Universel des Sciences Medicales,* tom. xii.

drive the blood against the contracted sides of the vessel.* Here the stream is retarded, and the artery, gradually giving way to the force employed, slowly enlarges, and degenerates into a true aneurism.

"An old woman, who had an incurvation of the spine and was lame, died in the hospital at Padua, about the middle of March, in the year 1747. She had been lately brought thither on account of a disorder of the apoplectic kind, which did not appear to have injured any other faculty but that of her speech. In the belly, the trunk of the great artery began, almost immediately after giving off the emulgents, to dilate itself gradually more and more the more it descended, till, a little above the division, it expanded itself wholly into an aneurism, which was of two inches diameter in every direction. From thence it was again gradually contracted; yet in such a manner, that the iliacs themselves appeared to be much wider than they naturally are, to a considerable extent. The internal surface of these vessels was unequal, but the internal surface of the aneurism still more so; where not only polypous concretions were found, but in one part of the coats bony concretions also. I should be inclined to suppose that the cause of these disorders of the aorta had, in great measure, consisted in the distorted figure of the spine; which, having a convexity in the thorax on the right side, had another on the left side, in the loins, which carried away the aorta along with it."†

When the arterial blood becomes in this manner diverted in its course, the circulation in the lower parts is diminished, and more of it flows upwards: hence arise apoplexies and palsies.

"In proportion as this depraved figure of the spine, which I have described, made an infliction upon the great artery that adhered thereto, so much did it resist the ready and easy flux of blood towards the inferior parts of the body. The consequence of this therefore was, that a greater quantity was sent from thence to the brain, whereby a disposition to a sanguineous apoplexy was brought on."‡

Luxations of the dorsal and lumbar vertebræ operate in the same way. They force the protruded bones upon the descending aorta, and squeeze its sides together, as we have already explained. This pressure, while it impedes the motion of blood towards the lower parts, drives an increase of it into other organs. In them the blood accumulates, the vessels are unduly distended, and their functions, whatever they may be, are dangerously oppressed. This preternatural fulness lays the

* Morgag. Epist. lxii. art. 11.

† Ibid. Epist. xxxviii. art. 40.

‡ Ibid. lxii. art. 12.

foundation of organic derangements, of an obstinate and dangerous character, in the head, chest, abdomen, and pelvis, according as the offending cause occupies an higher or lower station. A slight change in the direction of the luxated bones gives them a tendency towards the jugular veins; and thus, by interposing obstacles between the returning blood and the heart, the free course of the refluent stream is impeded. From this cause alone proceeds a most inveterate head-ach,* which is equally felt within and on the outside of the skull. When the impediment is of long continuance or operates severely, the blood-vessels of the brain become preternaturally distended: hence arise sanguineous apoplexy, paralytic seizures, epilepsy, lunacy, and various anomalous complaints in the head.

Luxations towards the right side of the back bring the dorsal and lumbar vertebræ into contact with the vena cava ascendens, the sides of which are pressed together, and in consequence all the superficial veins are seen to be turgid with blood. Morgagni relates the case of a mason, who, falling from an upper story of a house, and receiving a blow upon his loins, began to lose the sense of feeling in his feet, and to discharge the contents of the intestines and bladder involuntarily, and to have the other symptoms that are described. As this man died after four days, the three uppermost vertebræ of the loins were found to be so luxated as to be prominent a finger's-breadth into the cavity of the belly, and thereby to compress the large trunks of the vessels in such a manner as to bring their opposite parietes into mutual contact; from which it happened, amongst other things, that all the veins, from both extremities of the feet quite to the luxation, were turgid with blood, and hard, just as if they had been stuffed up by force."† The limbs feel stiff and uneasy in these cases. Soon afterwards, if the patient survive the accident, dropsical swellings take place, and keep gradually increasing. Persons of both sexes, in less violent attacks, and where the vertebral dislocation is slowly produced, become liable to hemorrhoids, and females to profuse menstruation and constant leucorrhea.

A curvature in the dorsal vertebræ, whether protuberant or lateral, generally displaces the liver and deranges its secretions. The ribs are forced upwards, and carry the liver along with them. This dislodgment is known by an hollowness in the back. The ribs become flat; the upper part of them is thrown outwards; and the liver can be distinctly felt below their under-edge. The blood, having to rise higher and perform a longer passage in the vena portarum to reach the liver, is impeded in its advance, and circulates through it with difficulty. The

* Cases 3d and 4th.

† Morgag. book iv. letter 56, art. 35.

biliary secretion is defective in quantity, and pale-coloured. The countenance assumes a sallow hue, the appetite fails, and the food is not properly digested. The difficulty which is felt in transmitting the blood through the liver, causes an accumulation of it in the whole system of the vena portarum. In consequence, the abdomen is tumid and painful;* the bowels are obstinately constipated, and hemorrhoids often prevail to a great degree.

A dislodgment of the other abdominal viscera disturbs their respective functions, and lays the foundation of bad health. An extraordinary and untenable opinion seems to prevail among the British faculty, in regard to lateral curvatures: according to them, persons labouring under this species of deformity are not to be reputed diseased, or to be supposed to suffer under any complaint in consequence of their shape. Medical men who inculcate such doctrines can have paid but little attention to the subject, or they would have discovered abundant reason for believing that not only delicate health, but a great variety of very obstinate and highly dangerous disorders, proceed directly from this source alone. The greatest vessels of the human body are firmly tied and bound closely down to the spine, within the neck, chest, and abdomen, so as to be forced to obey and follow every change of the vertebral column. Now, the first incurvation generally begins between the shouldors, because, in our different occupations, the right hand is chiefly employed. An obliquity of the spine in that particular part necessarily displaces not only the largest vessels of the body, but interferes directly and dangerously with the sanguineous circulation through the lungs and heart.† The returning blood, in the natural condition of the vena cava ascendens and descendens, glides along with an easy progression to the right auricle of the heart. From thence it makes the circuit of the lungs in the pulmonary vessels, and, having undergone its peculiar changes, is received into the left auricle and ventricle. It is driven from the latter into the aorta ascendens and descendens, to be distributed through every part of the body. The vigorous contractions of the heart to impel the blood may be distinctly felt, and the pulsatory vibrations of the arteries, at a great distance from the fountain of motion, show that the blood had been propelled into them with no small degree of effort. In the ordinary distribution of these vessels, the blood meets with no impediments to its course, and circulates freely; but, when the aorta and its dependent branches have been removed from their natural situation, things become very different. The blood, having to pass along distorted tubes, and encounter angles and prominences in its journey, becomes interrupted and hindered.

† Cases 2d, 3d, and 4th.

To overcome these impediments, the heart produces greater exertions, and palpitates violently. The circulation becomes forced, unnatural, irregular, and is carried on at an expense and effort of the heart, which waste its energies, and lay the foundation of the most untractable disorders. The unusual force employed drives the blood with additional impulse into the arteries. These become preternaturally distended, and the blood is driven with increased momentum against the tortuous sides of the canal, which gradually give way to the force employed, enlarge in their dimensions, and a portion of the blood is permanently retained in the vessel. This is a common origin of aneurismal swellings, and of vascular dilatations. In consequence of this interruption, the blood passes with increased difficulty out of the heart; which, from a curious provision of nature, increases in size and thickness, to be enabled to surmount the new obstacle. For the same reason, the pulmonary circulation is disturbed, and the blood accumulates in the vessels because it cannot get freely into the left auricle and ventricle. Nor is this all: the curvature between the shoulders diminishes the capacity of the chest, and, by its connection with the ribs, forces them and the sternum out of their proper situations. In this way the form of the breast is changed. The sides are unusually flat, and the breast-bone gets peaked. These alterations in the bony case reduce the internal dimensions of the chest: less room is allowed for the important functions of the heart and lungs; they are squeezed and compressed by firm and immovable parietes, in consequence of which the circulation is difficult and irregular. The lungs are imperfectly dilated, and a reduced supply of air is admitted into them at every inspiration. To compensate for a diminution in quantity, the respirations are quickened,* and the slightest exertions produce violent pantings, with a tickling cough, suffused countenance, and copious perspirations about the face and breast. This very dangerous state of things gives rise to tubercles and hemoptysis, which generally terminate in fatal consumptions.† Should the delicate invalid arrive at middle age, he is teazed with coughs, dyspnœa,‡ spasmodic asthma, and other distressing maladies, arising from thoracic compression and an interrupted circulation in the lungs and heart. Persons so situated are characterized by an haggard contracted countenance, and look older than their years warrant. Perpetually assaulted by one train of symptoms or another, they are soon worn out, and sink into the grave at an early age.

* De Haën.

† Lieutaud, De Haën.

‡ Lieutaud.

The inferior bend is generally fixed in the loins, and occupies a greater or smaller extent in different persons. The obliquity is usually towards the left side.* By retarding the arterial stream, it gives rise to aneurismal enlargements in an higher part of the aorta. When the progress of blood is diminished in the internal iliac arteries, amenorrhœa is a natural consequence.* The obstruction sometimes inclines towards the right side, and interrupts the refluent stream; thus giving rise to varicose veins, hemorrhoids, profuse menstruation, and uninterrupted leucorrhœa.† The lower curvature produces an obliquity in the pelvis, which forces down the right acetabulum.‡ This makes the corresponding leg andt high appear longer than their opposite fellows. The person becomes unsteady in his footsteps, and often stumbles in walking. This seeming difference in the length of the lower limbs deceives superficial observers into a belief that the patient labours under an affection of the hip-joint, when the true seat of the disorder is the spinal column.

When distortion is firmly established, the body generally ceases to grow in height or thickness: it either remains quite stationary, or actually gets more diminutive. The various progressive alterations which accompany its evolutions, and its advancement towards maturity, are unaccountably deferred. In males, the change of voice, the growth of beard, and other features of puberty, do not occur at the accustomed age. In females, though the menses burst forth prematurely and in abundance, the mammæ remain nearly quiescent, the eye continues dull, and the countenance is characterized by languor. The development of the constitution is equally marked in the change of pursuits and of objects to which it gives rise. The pleasures and recreations of the child are voluntarily relinquished, at a certain period of expansion, for those of manhood; but, when the natural propensities are unduly retarded by the causes enumerated, the frame and disposition of the mind continue juvenile and puerile to a late season.

This malconformation and diminution of stature lead to the destruction of health, the loss of beauty, of personal attractions, and of symmetrical proportion among the component members of the body, so much as to outweigh all the advantages of talents, high connections, and wealth.§

* Case 4th. † Ibid. ‡ Ibid.

§ The urgency for insertion of some other papers which we have had long in our possession, obliges us to defer the cases referred to in this article until the next Number.—Edit.

